# Effects of exogenous calcium additions on the ecological stoichiometric characteristics of various organs and soil nutrients and their internal stability in *Pinus tabuliformis*

**DOI:** 10.3389/fpls.2024.1428011

**Published:** 2024-09-05

**Authors:** Hui Li, Yaoyao Zhao, Xiaohang Weng, Yongbin Zhou, Yan Huo, Songzhu Zhang, Liying Liu, Jiubo Pei

**Affiliations:** ^1^ College of Forestry, Shenyang Agricultural University, Shenyang, Liaoning, China; ^2^ Research Station of Liaohe-River Plain Forest Ecosystem, Chinese Forest Ecosystem Research Network (CFERN), Shenyang Agricultural University, Changtu, China; ^3^ Institute of Modern Agricultural Research, Dalian University, Dalian, China; ^4^ Liaoning Dryland Agriculture and Forestry Research Institute, Chaoyang, Liaoning, China; ^5^ College of Land and Environment, Shenyang Agricultural University, Shenyang, Liaoning, China

**Keywords:** semi-arid region, exogenous calcium, *Pinus tabuliformis*, ecological stoichiometry, three north protected forests

## Abstract

**Introduction:**

*Pinus tabuliformis* as a crucial afforestation species in semi-arid regions, faces issues such as the reduction of plantations. Calcium plays a significant role in alleviating drought stress and promoting nutrient uptake in plants.

**Methods:**

Utilizing a pot experiment approach, seedlings were treated with exogenous calcium at five concentrations (0, 50, 100, 200, and 400 mg•kg-1). The nutrient content of the plants and soil was measured, and their ecological stoichiometric characteristics and internal stability were analyzed. This was followed by a series of related studies.

**Results:**

As the concentration of calcium increases, the contents of carbon, nitrogen, phosphorus, and potassium in various organs and the whole plant exhibit a trend of first increasing and then decreasing, peaking at calcium treatment of 50-100 mg•kg-1. Concurrently, the calcium concentration in plant organs and the entire plant gradually increases with the availability of calcium in the soil. The addition of exogenous calcium has a certain impact on the ecological stoichiometric ratios (C:N, C:P, N:P) of *Pinus tabuliformis* seedlings' leaves, stems, roots, and the whole plant, exhibiting distinct variation characteristics. At calcium concentrations of 50-100 mg•kg-1, the ratios of C:N and C:P are relatively lower. Under calcium concentrations of 0, 50, and 100 mg•kg-1, soil calcium shows a positive correlation with the total carbon (TC), total nitrogen (TN), total phosphorus (TP), total potassium (TK), and calcium contents in leaves, stems, roots, and the entire plant. However, at calcium concentrations of 200 and 400 mg•kg-1, soil calcium exhibits a significant positive correlation with the calcium content in leaves, stems, roots, and the entire plant, and a significant negative correlation with the total phosphorus, total nitrogen, total phosphorus, and total potassium contents. After the addition of exogenous calcium at different concentrations, most stoichiometric indices of various organs of *Pinus tabuliformis* seedlings demonstrate strong balance.

**Discussion:**

Calcium, as an essential structural component and second messenger, regulates the nutrient uptake and utilization in plants, influencing the stoichiometry. However, both low and high concentrations of calcium can be detrimental to plant growth by disrupting nutrient metabolism and internal structures. Consequently, there exists an optimal calcium concentration for nutrient absorption.

## 1 Introduction

The semi-arid region is characterized by a dry climate, scarce precipitation, and an extremely fragile ecological environment. Planted forests play a crucial role in this area by helping to prevent winds, fix sands, and maintain soil and water (Barroso et al., 2022). *Pinus tabuliformis* is one of the main afforestation species in semi-arid regions of China and is also the main component of the forests in the western part of Liaoning province. Its exceptional drought-resistant and indigenous characteristics have also made it useful in other projects to prevent desertification and control sandstorms, such as the “Three-North Protected Forest Program” and the “Returning Cultivated Land to Forests Project” (Zhu and Zheng, 2019; Zeng et al., 2020). Despite its ecological significance, *Pinus tabuliformis* has faced significant challenges in recent decades, experiencing serious decline and mortality issues. Furthermore, plantation forests have also triggered other aspects of ecological crises due to monoculture stand structure and poor stand stability, including declining land productivity, increasing pests and diseases incidence, and declining carbon storage (Liu et al., 2019; Li et al., 2020; Zhang et al., 2021). Therefore, scientific and systematic research on the environmental adaptation of plantation forest growth in semi-arid areas, elimination and mitigation of constraints leading to the decline of plantation forests have become the key to solving the problem of healthy development of plantation forests in the region, and also the way to the development of healthy and sustainable management of plantation forests in the region.

The defining element that migrates within the soil of semi-arid regions is calcium. In these areas, the soil is predominantly sandy and nutrient-poor, exhibiting a deficiency in calcium. While on the other hand, due to the seasonal fluctuations in rainfall and drought typical of semi-arid environments, Ca^2+^ leaching within the soil can precipitate at certain depths, forming a layer of calcium accumulation that influences the soil’s physical, chemical, and biological characteristics (Arkley, 1963; Certini and Scalenghe, 2006; Ermakov et al., 2020; Lawrence et al., 2020; Sun et al., 2021). Calcium plays a pivotal role in plant growth, moderate levels of calcium can mitigate drought stress and foster growth, while a deficiency can inhibit growth (Aras et al., 2021; Li et al., 2022a, b). Studies in *Pinus massoniana* have shown that calcium deficiency significantly inhibits seedling growth and development and impairs photosynthesis processes (Hu et al., 2022). Therefore, from the perspective of element utilization in soil-forest vegetation systems, calcium can be considered an indirect influencing factor on forest vegetation growth. Based on this, in environments where vegetation growth in semi-arid regions is affected by both calcium deficiency and abundance, it is necessary to conduct in-depth research on the different effects and mechanisms of calcium regulation in artificial forests in this region. This is of significant importance for studying the mechanisms of decline in artificial forests in semi-arid regions and addressing ecological issues and research needs in artificial forest cultivation in these areas.

Since the publication of Sterner and Elser (2002), the field of ecological stoichiometry has matured considerably over time and has emerged as a new approach to exploring the effects of environmental nutrient supply fluctuations on nutrient element limitations, population dynamics, and ecosystem functioning (Qi et al., 2020; Li et al., 2023b). It primarily investigates the coupled relationships among the essential nutrients for plant growth, C, N, P, and K, reflecting the balance of resource acquisition by plants and their growth strategies (Liao et al., 2023; Wei et al., 2021a). The ecological stoichiometric characteristics of plant C:N:P can indicate the patterns of C accumulation and N, P limitations in the ecosystem they inhabit (Elser et al., 2010). Nutrient elements in plants are inevitably intertwined with soil nutrients, and measuring nutrients in the soil is a direct method to explore soil nutrient supply capacity (Chapin et al., 1986). Soils and their nutrients provide a favorable growth environment for plant growth, while plants, in turn, impact the structure of soil nutrients. The interaction between plants and soil constitutes the foundation of plant-soil feedback (Liu et al., 2023). Ecological stoichiometry is also considered an effective new approach to exploring soil-plant interactions and the cycling characteristics of ecosystem elements (Wu et al., 2023).

Previous studies on the effects of exogenous nutrient addition on the stoichiometric characteristics of plants have focused on elements such as silicon and nitrogen, but there is a paucity of research on the impacts of exogenous calcium addition (Long et al., 2018; Xu et al., 2021). Past studies on *Pinus tabuliformis* have primarily examined aspects like forest management, water regulation, and site quality, while the response of *Pinus tabuliformis* to soil calcium and the underlying physiological regulation mechanisms remain underexplored. Therefore, this study used *Pinus tabuliformis* seedlings as the research subject, applying different exogenous calcium concentration treatments. Targeting the calcium-deficient and calcium-rich soil environments typical of the semi-arid regions of the Three-North Shelter Forest in China, the study investigated the stoichiometric characteristics of different organs of *Pinus tabuliformis* seedlings. The aim was to identify the optimal calcium concentration for *Pinus tabuliformis* growth, further complement the research on the impacts of calcium on forest stands, and provide scientific theoretical support for the rational cultivation and healthy, sustainable management of artificial forests in semi-arid regions from the perspective of nutrient absorption.

## 2 Materials and methods

### 2.1 Experimental design

The experiment was conducted at No.37 experimental shed in Beishan Research Base of Shenyang Agricultural University. Three-year-old *Pinus tabuliformis* seedlings with consistent growth were chosen as the test materials. The collected sandy soil was carefully sieved to eliminate stones and impurities. Then, 3 kg of the air-dried soil weight was precisely weighed and mixed with 2 kg of 60-mesh quartz sand to create the potting soil. After thoroughly mixing the substrate, the soil was rinsed with a diluted hydrochloric acid solution to eliminate any potential Ca^2+^ ions before being filled into the pots. The pH of the soil-sand mixture after the hydrochloric acid wash was measured at 6.3. The seedlings, along with nutrient pots, were planted in plastic pots with an inner diameter of 23.8 cm, a depth of 24.8 cm, and a volume of approximately 11.36 L, in June 2019. Each pot received one seedling, and a plastic tray was placed beneath the pot to prevent water loss. After a two-week period of seedling acclimatization, a calcium-deficient standard nutrient solution was uniformly added once. This nutrient solution was prepared using ultrapure water according to Xie (2014) recipe for sand cultivation nutrient solution. The pH of the nutrient solution was adjusted to a range of 5-6 using NaOH. The components of the nutrient solution included 5 ml/L KNO_3_, 5 ml/L MgSO_4_, 5 ml/L KH_2_PO_4_, 5 ml/L NaNO_3_, and 5 ml/L EATA-Fe. Trace elements such as H_3_BO_3_, MnCl_2_, CuSO_4_, ZnSO_4_, and H_2_MoO_4_ were also incorporated into the nutrient solution.

In this experiment, five different treatments were set up and each treatment was replicated six times. Five calcium concentration levels of 0, 50, 100, 200 and 400 mg·kg^-1^ were set according to Paiva et al., 1998 and the condition that the local water-soluble calcium content ranged from 14.66 ~ 299.02 mg·kg^-1^ (Zhou and Zou, 2017). Treatments were applied in July and calcium was supplied by anhydrous CaCl_2_.To ensure the even distribution of CaCl_2_ in the soil, the CaCl_2_ solution was divided into aliquots and then irrigated into the soil. The experimental sandy soil was sourced from the understory soil of *Pinus tabuliformis* forest in Fujia Forest Farm, Changtu County, Tieling City, Liaoning Province. The soil samples were randomly collected from multiple points under standard standing conditions, with a collection depth ranging from 0-40 cm. Throughout the testing period, measures were taken to closely monitor the experimental phenomena. Adequate moisture was maintained by using deionized water, and necessary care, including loosening and weeding, was undertaken to ensure the smooth progress of the experiment.

### 2.2 Determination indicators and methods

In June of the following year, seedlings and soil samples were harvested for analysis. The seedlings were carefully washed and separated into leaves, stems, and roots. They were then heated at 105 °C for 0.5 hours and subsequently dried at 65 °C until a constant weight was achieved. The dry weight of each plant part was measured using a precision balance with a sensitivity of 1/1000. The individual dry weights of the leaves, stems, and roots were combined to obtain the total biomass of the seedlings. The pulverized leaves, stems, and roots were further crushed using a ball mill until they could pass through a 100-mesh sieve. These samples were then stored in small envelopes under dry conditions. As for the soil samples, they were air-dried, filtered, and then crushed and ground using a pulverizer. The final samples were stored in self-sealing bags and kept dry (Weng et al., 2022).

The determination of total carbon (TC) and total nitrogen (TN) contents in the plant leaves, stems, and roots was conducted using an elemental analyzer following the method described by Ding et al. (2022). The total phosphorus (TP) content in the plant tissues was determined using the acid-soluble molybdenum-antimony colorimetric method, as outlined by da Silveira Sousa Junior et al. (2022). The total potassium (TK) and calcium (Ca) contents in the plant tissues were determined using the acid-soluble-flame photometric method, following the procedure described by Sarto et al. (2019). For the soil samples, the total carbon (TC) and total nitrogen (TN) contents were determined using an elemental analyzer. The soil’s total phosphorus (TP) content was determined through acid solubility-molybdenum-antimony colorimetry. The soil available potassium (AK) content was determined using the NH_4_OAc extraction-flame photometric method, as described by Yin et al. (2021). The soil water-soluble calcium and soil-exchangeable calcium contents were determined following the method developed by Zhou Wei and Lin Bao, as mentioned by He et al. (2012).

### 2.3 Statistical analysis

The chemical stoichiometric characteristics of *Pinus tabuliformis* seedlings were subjected to Pearson correlation analysis with soil. When the test results were significant (p<0.05), an internal homeostasis model was introduced for further quantitative analysis of the stoichiometric characteristics of seedling leaves, stems, roots, and whole plants in response to environmental changes concerning the content and ratios of C, N, P, K, and Ca elements. The equation of the internal homeostasis model is: $y=cx^{1/H}\;$ (Sterner and Elser, 2002). In this equation, y represents the nutrient elements C, N, P, K, and Ca content and ratios in different plant organs, x represents the corresponding nutrient element content and ratios in the soil environment, c is a constant, and H is the internal homeostasis index. The strength of internal homeostasis is measured by 1/H (Hood and Sterner, 2010). If the fitting equation does not reach a significant level (*p*≥0.05) or 1/H ≤ 0, it is strictly homeostatic; 0<1/H<0.25, it is homeostatic; 0.25<1/H<0.5, it is weak homeostatic; 0.5<1/H<0.75, it is weak responsive; 1/H >0.75, it is responsive (Persson et al., 2010).

Basic data organization was done using Microsoft Excel 2019 software, significance testing (*p*<0.05) was performed using SPSS 19, redundancy analysis and plotting were conducted using Canoco 5 software, and plotting was carried out using GraphPad Prism 8.0.2.

## 3 Results

### 3.1 Effects of exogenous calcium addition on the nutrient content of various organs and the distribution characteristics of nutrient content in *Pinus tabuliformis* seedlings

**Figure 1:**
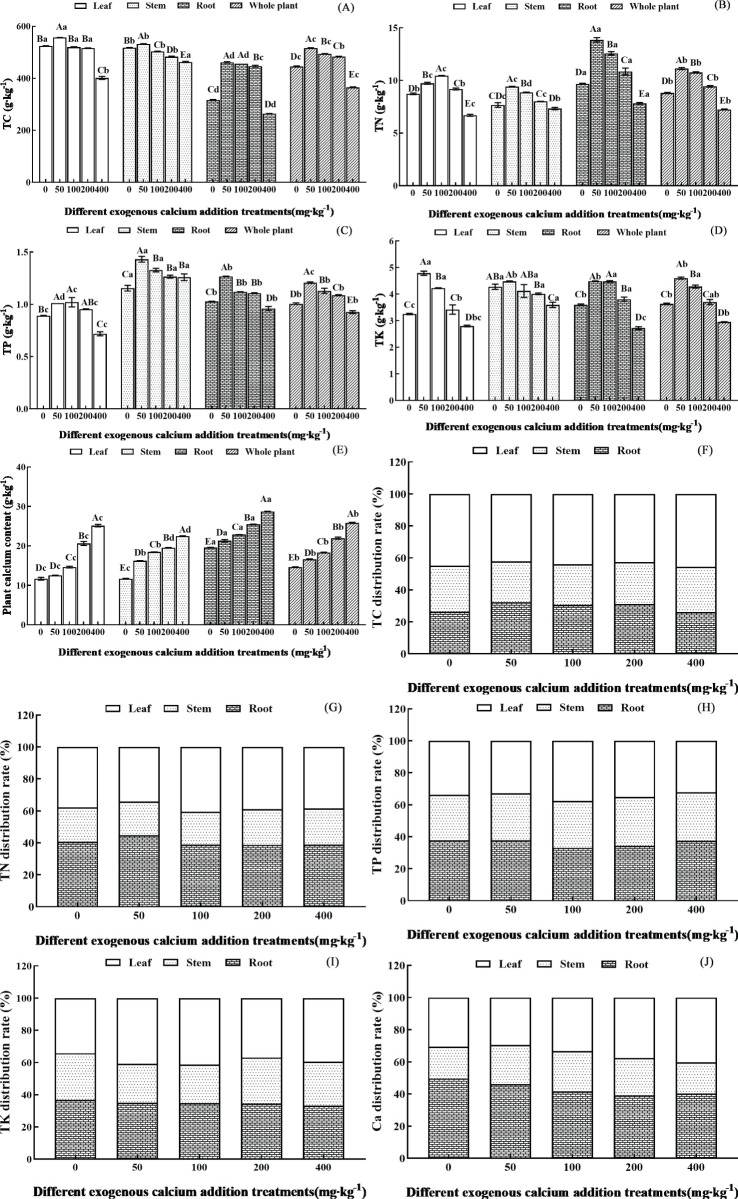
Nutrient content and distribution characteristics of different organs of *Pinus tabuliformis* seedlings under exogenous calcium supplementation. Capital letters indicated the difference between different calcium concentrations in leaves, stems, roots and whole plants (*p*<0.05), while lower letters indicated the difference between leaves, stems, roots and whole plants at the same concentration (*p*<0.05). **(A)** TC (total carbon); **(B)** TN(total nitrogen); **(C)** TP (total phosphorus); **(D)** TK (total potassium); **(E)** Plant calcium content; **(F)** TC distribution rate; **(G)** TN distribution rate; **(H)** TP distribution rate; **(I)** TK distribution rate; **(J)** Ca distribution rate.

As shown in **Figures 1A-E**, the exogenous calcium addition significantly influenced the contents of TC, TN, TP, TK, and Ca in the leaves, stems, roots, and whole seedlings of *Pinus tabuliformis* (*p*<0.05). The contents of TC, TN, TP, and TK in different organs and the whole seedling showed a trend of first increasing and then decreasing with the increase of calcium concentration, while the Ca content gradually increased.

The TC content in the leaves, stems, and whole plants reached the maximum value under the treatment of 50 mg·kg^-1^ calcium, increasing by 6.18%, 2.85%, and 15.82% respectively compared to the control without calcium; while the root content reached the highest at calcium concentrations of 50-100 mg·kg^-1^, which increased by 44.63% compared to the control without calcium. The TN and TP content in the leaves significantly exceeded other treatments at 100 mg·kg^-1^, increasing by 19.69% and 14.03%, respectively, compared to the control without calcium; while the stem, root, whole plant was significantly higher at 50 mg·kg^-1^, with increases of 22.75% and 23.91%, 43.08% and 23.41%, 26.25% and 19.95% respectively, when compared to no calcium application (*p*< 0.05). The TK content in the leaves, stems, and whole plants reached its peak at a calcium treatment of 50 mg·kg^-1^, with increases of 47.27%, 4.75%, and 26.66% respectively, compared to no calcium, while the TK content in the roots peaked at a calcium treatment of 100 mg·kg^-1^, showing an increase of 24.60% over the absence of calcium. Subsequently, with the increase of calcium concentration, the TC, TN, TP, and TK contents in each organ and the whole plant showed a decreasing trend, and when the calcium concentration reached 400 mg·kg^-1^, they all significantly decreased compared to the control without calcium (except for the TP content in stems) (*p*< 0.05). The calcium content in each organ and the whole plant gradually increased with the increase of exogenous calcium concentration. When the calcium concentration increased to 400 mg·kg^-1^, the calcium contents of the leaves, stems, roots, and whole plants significantly increased by 115.01%, 92.61%, 46.76%, and 76.87%, respectively, compared to the control without calcium (*p*< 0.05).

Overall, the contents of TC, TN, TP, TK, and Ca in *Pinus tabuliformis* seedlings exhibited the order of TC > Ca > TN > TK > TP. The contents of other elements except for calcium reached the maximum value when the calcium concentration was between 50 and 100 mg·kg^-1^.

The nutrient content (unit: g) distribution characteristics of leaves, stems, and roots are shown in **Figures 1F-J**. Under the same calcium concentration, significant differences were observed in the contents of TC, TN, TP, TK, and Ca in different organs of *Pinus tabuliformis* (*p<* 0.05). The content of TC and TK was characterized by leaves > roots > stems, while the content of TN, TP, and Ca was characterized by roots > leaves > stems. In terms of the average proportion of each element in different organs, C and K elements were most distributed in leaves, accounting for 43.88% and 38.51% respectively, while N, P, and Ca elements were most distributed in roots, accounting for 40.40%, 36.07%, and 43.33%, respectively.

### 3.2 Effect of exogenous calcium addition on ecological stoichiometric ratios of various organs of *Pinus tabuliformis* seedlings

**Figure 2:**
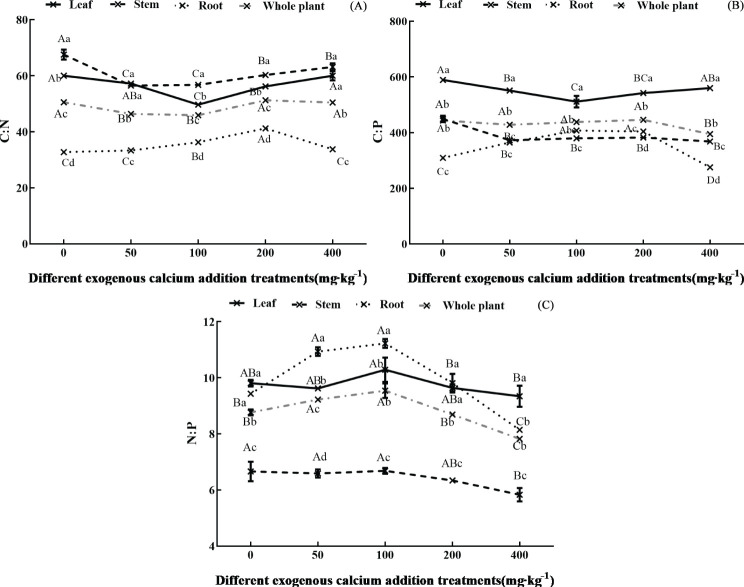
Ecological stoichiometric ratio of organs of *Pinus tabuliformis* seedlings under exogenous calcium supplementation. Capital letters indicated the difference between different calcium concentrations in leaves, stems, roots and whole plants (*p*<0.05), while lower letters indicated the difference between leaves, stems, roots and whole plants at the same concentration (*p*<0.05). **(A)** C:N; **(B)** C:P; **(C)** N:P.

The ecological stoichiometric ratios (C:N, C:P, N:P) of different organs of *Pinus tabuliformis* seedlings under varying calcium concentrations are shown in **Figures 2A-C**. The addition of exogenous calcium significantly affected the leaf C:N, C:P, and N:P (except N:P) (*p*<0.05), presenting a “decreasing-increasing”, “decreasing-increasing”, and “increasing-decreasing” trend, respectively, with the gradual increase in added calcium concentration. The leaf C:N and C:P reached their minimum values at 100 mg·kg^-1^ calcium treatment. The calcium addition also significantly affected the stem C:N, C:P, and N:P (except N:P) (*p*<0.05), showing a “decreasing-increasing”, “decreasing”, and “decreasing” trend, respectively, with the gradual increase in added calcium concentration. The stem C:N was significantly reduced and reached the minimum at 50-100 mg·kg^-1^ calcium treatment compared to the control. The root C:N, C:P, and N:P were significantly affected by calcium addition (*p*<0.05), all exhibiting an “increasing-decreasing” trend with increasing calcium concentration, with the ratios ranging from 32.10-42.78, 267.25-412.12, and 8.01-11.51, respectively. The whole plant C:N, C:P, and N:P were also significantly affected by calcium (*p*<0.05), showing a “decreasing-increasing”, “decreasing”, and “increasing-decreasing” trend with increasing calcium concentration, respectively. For the whole plant, the C:N was significantly reduced and reached the minimum at 100 mg·kg^-1^ calcium, and the N:P was significantly increased and reached the maximum at 50-100 mg·kg^-1^ calcium compared to the control.

The stoichiometric ratios differed among organs, with the overall trend being stem > leaf > whole plant > root for C:N, leaf > whole plant > stem > root for C:P, and leaf, stem, root, and whole plant showing little difference for N:P.

### 3.3 Effect of exogenous calcium on soil nutrient content and ecological stoichiometric ratios of *Pinus tabuliformis* seedlings

**Table 1** presents the values of soil nutrient contents and stoichiometric ratios of *Pinus tabuliformis* seedlings under different calcium concentrations. With the increase of calcium concentration, soil contents of TC, TN, TP, AK, C:N, C:P, and N:P showed no significant differences (*p*>0.05), while soil water-soluble calcium and exchangeable calcium exhibited significant differences and showed a trend of gradually increasing content (*p*<0.05).

**Table 1:** Soil elemental contents and ecological stoichiometric ratios of *Pinus tabuliformis* seedlings under exogenous calcium addition.

Calcium concentration (mg·kg^-1^)	Total carbon (g·kg^-1^)	Total nitrogen (g·kg^-1^)	Total phosphorus (g·kg^-1^)	Available potassium (g·kg^-1^)	Water soluble calcium (g·kg^-1^)	Exchange of calcium (g·kg^-1^)	C:N	C:P	N:P
0	4.23 ± 0.03a	0.49 ± 0.01b	0.15 ± 0.01a	0.12 ± 0.01a	0.12 ± 0.005d	1.73 ± 0.04d	8.63 ± 0.15ab	28.77 ± 1.12a	3.33 ± 0.07ab
50	4.53 ± 0.26a	0.62 ± 0.01a	0.15 ± 0.01a	0.12 ± 0.002a	0.27 ± 0.004c	1.76 ± 0.05cd	7.37 ± 0.53b	31.00 ± 0.59a	4.26 ± 0.39a
100	3.72 ± 0.31a	0.47 ± 0.08b	0.14 ± 0.00a	0.13 ± 0.003a	0.30 ± 0.002c	1.83 ± 0.02c	8.11 ± 0.66ab	25.96 ± 2.21a	3.29 ± 0.53ab
200	4.16 ± 0.34a	0.47 ± 0.02b	0.15 ± 0.00a	0.13 ± 0.001a	0.34 ± 0.021b	2.34 ± 0.002b	8.91 ± 0.34a	27.97 ± 2.33a	3.13 ± 0.14b
400	4.54 ± 0.10a	0.57 ± 0.01ab	0.15 ± 0.00a	0.12 ± 0.001a	0.54 ± 0.009a	3.19 ± 0.02a	8.03 ± 0.03ab	29.45 ± 0.12a	3.67 ± 0.03ab

small letters indicate the difference between different concentrations (p<0.05).

After the application of exogenous calcium, the soil nutrient contents changed as follows: TC (3.18 g·kg^-1^ to 4.98 g·kg^-1^), TN (0.34 g·kg^-1^ to 0.63 g·kg^-1^), TP (0.13 g·kg^-1^ to 0.17 g·kg^-1^), AK (0.10 g·kg^-1^ to 0.14 g·kg^-1^), water-soluble calcium (0.11 g·kg^-1^ to 0.55 g·kg^-1^), and exchangeable calcium (1.66 g·kg^-1^ to 3.21 g·kg^-1^). Without adding exogenous calcium, the content of water-soluble calcium and exchangeable calcium was 0.12 g·kg^-1^ and 1.73 g·kg^-1^ respectively. As the calcium concentration gradually increased, their content significantly increased by 129.21% and 1.75%, 149.58% and 6.17%, 190.32% and 35.82%, 353.03% and 84.56% (*p*<0.05) respectively.

After the application of exogenous calcium, the range of soil C:N ratios changed from 6.46 to 9.48, C:P ratios changed from 22.13 to 32.07, and N:P ratios changed from 2.37 to 4.96. Specifically, at a calcium concentration of 200 mg·kg^-1^, the soil C:N ratio was maximum (8.91), and at 50 mg·kg^-1^, it was minimum (7.37); at a calcium concentration of 50 mg·kg^-1^, the soil C:P ratio was maximum (31.00), and at 100 mg·kg^-1^, it was minimum (25.96); at a calcium concentration of 50 mg·kg^-1^, the soil N:P ratio was maximum, at 4.26.

### 3.4 Effect of exogenous calcium on the correlation between seedling-soil ecological stoichiometric traits of *Pinus tabuliformis* seedlings

The **Table 2** shows the correlation coefficients between the nutrient content and stoichiometric ratios of leaves, stems, roots, and whole seedlings of *Pinus tabuliformis* and the soil. The analysis indicates that the chemical stoichiometric characteristics of *Pinus tabuliformis* seedlings are correlated with the soil.

**Table 2:** Correlation coefficient between nutrient content and stoichiometric ratio of leaves, stems, roots and whole plants of *Pinus tabuliformis* seedlings and soil.

Calcium concentration	Plant indicators		Soil indicators							
			TC	TN	TP	AK	Ca	C:N	C:P	N:P
0、50、100 mg·kg^-1^	TC	Leaf	0.548	0.649	0.048	-0.03	0.036	-0.442	0.582	0.583
		Stem	0.615	0.599	0.03	-0.094	-0.283	-0.317	0.676*	0.548
		Root	-0.096	0.305	-0.132	0.218	0.850**	-0.508	-0.022	0.329
		Whole plants	0.089	0.447	-0.103	0.201	0.723*	-0.548	0.167	0.45
	TN	Leaf	-0.354	-0.047	-0.027	0.214	0.849**	-0.23	-0.392	-0.039
		Stem	0.123	0.46	-0.185	0.364	0.754*	-0.537	0.26	0.488
		Root	0.198	0.504	0.012	0.14	0.701*	-0.547	0.203	0.451
		Whole plants	0.061	0.397	-0.041	0.202	0.786*	-0.51	0.085	0.373
	TP	Leaf	-0.405	-0.163	-0.106	0.407	0.661	-0.057	-0.387	-0.101
		Stem	0.168	0.395	0.189	-0.028	0.534	-0.408	0.044	0.29
		Root	0.346	0.617	0.018	0.033	0.473	-0.578	0.371	0.561
		Whole plants	0.068	0.333	0.037	0.129	0.574	-0.389	0.043	0.295
	TK	Leaf	0.111	0.505	-0.164	0.191	0.722*	-0.616	0.237	0.528
		Stem	0.759*	0.932**	0.114	-0.598	-0.059	-0.757*	0.779*	0.805**
		Root	-0.14	0.249	-0.105	0.201	0.834**	-0.462	-0.09	0.268
		Whole plants	0.147	0.557	-0.117	0.087	0.732*	-0.671*	0.244	0.555
	Ca	Leaf	-0.445	-0.169	-0.232	0.454	0.866**	-0.14	-0.35	-0.081
		Stem	-0.338	0.053	-0.156	0.263	0.913**	-0.357	-0.278	0.104
		Root	-0.336	-0.075	0.01	0.241	0.832**	-0.176	-0.401	-0.083
		Whole plants	-0.375	-0.032	-0.141	0.308	0.906**	-0.27	-0.334	0.014
	C:N	Leaf	0.511	0.259	0.032	-0.19	-0.770*	0.067	0.564	0.234
		Stem	0.056	-0.294	0.219	-0.451	-0.879**	0.455	-0.08	-0.345
		Root	-0.84**	-0.428	-0.398	0.271	0.742*	-0.082	-0.65	-0.227
		Whole plants	0.009	-0.285	-0.019	-0.208	-0.828**	0.421	0.043	-0.238
	C:P	Leaf	0.642	0.412	0.14	-0.441	-0.719*	-0.086	0.631	0.319
		Stem	0.024	-0.252	-0.165	0.029	-0.662	0.373	0.155	-0.167
		Root	-0.421	-0.027	-0.208	0.304	0.926**	-0.314	-0.334	0.051
		Whole plants	0.029	0.064	-0.367	0.121	-0.02	-0.12	0.299	0.187
	N:P	Leaf	0.044	0.173	0.115	-0.285	0.348	-0.28	-0.041	0.092
		Stem	-0.067	0.071	-0.693*	0.785*	0.335	-0.152	0.415	0.319
		Root	-0.045	0.263	-0.027	0.255	0.864**	-0.417	-0.048	0.237
		Whole plants	0.011	0.34	-0.179	0.266	0.856**	-0.515	0.124	0.357
200、400 mg·kg^-1^	TC	Leaf	-0.506	-0.920**	-0.757	0.915*	-0.997**	0.784	-0.314	-0.889*
		Stem	-0.591	-0.879*	-0.487	0.853*	-0.947**	0.6	-0.494	-0.925**
		Root	-0.443	-0.884*	-0.703	0.948**	-0.997**	0.814*	-0.263	-0.862*
		Whole plants	-0.48	-0.902*	-0.71	0.934**	-0.999**	0.791	-0.301	-0.882*
	TN	Leaf	-0.515	-0.898*	-0.629	0.922**	-0.992**	0.737	-0.366	-0.903*
		Stem	-0.51	-0.922**	-0.886*	0.847*	-0.954**	0.778	-0.278	-0.851*
		Root	-0.302	-0.814*	-0.732	0.972**	-0.974**	0.901*	-0.099	-0.768
		Whole plants	-0.424	-0.877*	-0.715	0.952**	-0.995**	0.829*	-0.238	-0.849*
	TP	Leaf	-0.483	-0.914*	-0.8	0.910*	-0.989**	0.805	-0.276	-0.868*
		Stem	0.538	0.314	0.567	0.339	-0.046	0.268	0.411	0.203
		Root	-0.571	-0.951**	-0.849*	0.846*	-0.970**	0.738	-0.356	-0.897*
		Whole plants	-0.451	-0.896*	-0.787	0.921**	-0.983**	0.822*	-0.245	-0.851*
	TK	Leaf	0.027	-0.574	-0.573	0.979**	-0.854*	0.980**	0.213	-0.524
		Stem	-0.642	-0.959**	-0.916*	0.732	-0.906*	0.646	-0.414	-0.885*
		Root	-0.314	-0.802	-0.627	0.985**	-0.978**	0.867*	-0.145	-0.786
		Whole plants	-0.249	-0.775	-0.679	0.987**	-0.963**	0.915*	-0.057	-0.737
	Ca	Leaf	0.676	0.979**	0.742	-0.820*	0.973**	-0.635	0.506	0.965**
		Stem	0.477	0.904*	0.731	-0.932**	0.998**	-0.799	0.291	0.878*
		Root	0.523	0.919**	0.703	-0.917**	0.998**	-0.758	0.35	0.905*
		Whole plants	0.593	0.950**	0.725	-0.879*	0.993**	-0.708	0.42	0.935**
	C:N	Leaf	0.35	0.565	0.032	-0.734	0.724	-0.436	0.374	0.684
		Stem	0.356	0.759	0.991**	-0.644	0.747	-0.723	0.076	0.616
		Root	-0.642	-0.939**	-0.611	0.848*	-0.972**	0.622	-0.51	-0.958**
		Whole plants	-0.823*	-0.8	-0.396	0.418	-0.668	0.133	-0.777	-0.854*
	C:P	Leaf	0.323	0.758	0.980**	-0.694	0.776	-0.771	0.043	0.62
		Stem	-0.873*	-0.876*	-0.812*	0.298	-0.614	0.179	-0.7	-0.815*
		Root	-0.391	-0.846*	-0.644	0.967**	-0.990**	0.828*	-0.224	-0.834*
		Whole plants	-0.498	-0.871*	-0.582	0.911*	-0.978**	0.716	-0.361	-0.885*
	N:P	Leaf	-0.204	-0.196	0.419	0.367	-0.329	0.044	-0.358	-0.374
		Stem	-0.663	-0.882*	-0.964**	0.512	-0.736	0.486	-0.421	-0.775
		Root	-0.107	-0.674	-0.606	0.996**	-0.917*	0.951**	0.076	-0.636
		Whole plants	-0.378	-0.822*	-0.583	0.965**	-0.981**	0.807	-0.229	-0.824*

**Significant correlation at 0.01 level (bilateral).

*Significant correlation at 0.05 level (bilateral).

TC, total carbon; TN, total nitrogen; TP, total phosphorus; TK, total potassium; AK, available potassium; Ca, calcium.

At calcium concentrations of 0, 50, and 100 mg·kg^-1^, soil Ca is mostly positively correlated with the nutrient (TC, TN, TP, TK, Ca) contents in leaves, stems, roots, and whole seedlings, and negatively correlated with the C:N and C:P ratios in leaves, stems, and whole seedlings, but positively correlated with the N:P ratios in leaves, stems, roots, and whole seedlings. At calcium concentrations of 200 and 400 mg·kg^-1^, soil Ca is positively correlated with the Ca contents in leaves, stems, roots, and whole seedlings, but negatively correlated with the TC, TN, TP, and TK contents in leaves, stems, roots, and whole seedlings, and negatively correlated with the C:N, C:P, and N:P ratios in roots, but the correlations with the C:N, C:P, and N:P ratios in leaves and stems are not significant.

The homeostasis index (1/H) calculations show that at calcium concentrations of 0, 50, and 100 mg·kg^-1^, the Ca in leaves, stems, roots, and whole seedlings exhibits “sensitive” homeostasis. At calcium concentrations of 200 and 400 mg·kg^-1^, the N in leaves, stems, roots, and whole seedlings, and the P in roots exhibit “strict homeostasis”, while the K in leaves, roots, and whole seedlings exhibits “sensitive” homeostasis, and the Ca in leaves, stems, roots, and whole seedlings exhibits “weakly sensitive” to “weakly homeostatic” homeostasis. The whole seedling N:P ratio also exhibits “strict homeostasis”, and the other elements in different organs generally exhibit “strict homeostasis”.

### 3.5 Response of ecological stoichiometric characteristics of *Pinus tabuliformis* seedlings to environmental factors under exogenous calcium supplementation

**Figure 3:**
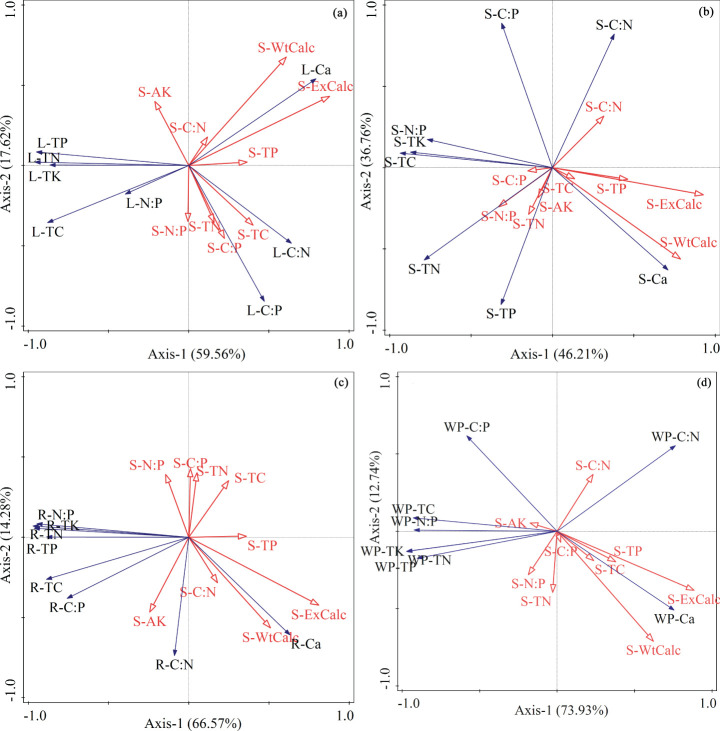
Two-dimensional ordination diagram of redundancy analysis of ecological stoichiometric characteristics and soil factors of *Pinus tabuliformis* seedlings. The blue solid arrow represents the elemental indicators of each organ, while the red hollow arrow represents soil factors. **(A)** Leaf; **(B)** Stem; **(C)** Root; **(D)** Whole plant; TC, total carbon; TN, total nitrogen; TP, total phosphorus; TK, total potassium; Ca, calcium; AK, available potassium; ExCalc, Exchange calcium; WtCalc, Water-soluble calcium.

As shown in the redundancy analysis (RDA) two-dimensional ordination diagram (**Figure 3**), the first two axes effectively demonstrate the relationship between soil factors and the stoichiometric characteristics of leaves, stems, roots, and whole plants of oil pine seedlings. The first two axes of **Figure 3** (a, b, c, and d) explain a significant portion of the variation in ecological stoichiometric characteristics. Specifically, they account for 77.19%, 82.96%, 80.85%, and 86.67% of the variation in the characteristics of leaves, stems, roots, and whole plants of *Pinus tabuliformis* seedlings, respectively. Furthermore, an analysis was conducted to evaluate the importance of soil environmental factors in contributing to the chemometric traits of the seedlings (see **Table 3**). The findings indicate that each soil factor has a varying contribution to the different chemometric traits of *Pinus tabuliformis* seedlings. Broadly speaking, the stoichiometric characteristics of *Pinus tabuliformis* seedlings are primarily influenced by the exchange calcium and water-soluble calcium, which have reached a highly significant level (*p*< 0.01). Additionally, the stoichiometric characteristics of *Pinus tabuliformis* leaves and stems are also influenced by soil TN and AK and N:P at a highly significant level (*p*< 0.01), with contribution rates of 10.3%, 6.8%, and 6.3%, respectively. The remaining soil factors have lower contribution rates.

**Table 3:** The importance order and significance test results of environmental factors of *Pinus tabuliformis* seedlings.

	Leaf				Stem			
Importance order	Soil factors	Contribution/%	F value	P value	Soil factors	Contribution/%	F value	P value
1	S-exchange calcium	57.1	12.7	0.002	S-exchange calcium	43.9	9	0.002
2	S-water- soluble calcium	23	7.8	0.002	S-water- soluble calcium	37.2	16.8	0.002
3	S-TN	10.3	4.5	0.004	S-AK	6.8	3.8	0.004
4	S-C:P	3.7	1.7	0.218	S-N:P	6.3	4.7	0.004
5	S-AK	2	0.9	0.402	S-TP	2.2	1.8	0.158
6	S-TP	1.9	0.8	0.46	S-C:N	1.5	1.1	0.352
7	S-N:P	0.8	0.4	0.702	S-TN	1.3	1.1	0.392
8	S-TC	0.7	0.3	0.794	S-C:P	0.5	0.4	0.79
9	S-C:N	0.4	0.1	0.928	S-TC	0.3	0.2	0.912
	Root				Whole plant			
Importance order	Soil factors	Contribution/%	F value	P value	Soil factors	Contribution/%	F value	P value
1	S-exchange calcium	53.9	11.6	0.002	S-exchange calcium	64.6	19.4	0.002
2	S-water- soluble calcium	26.2	9.1	0.006	S-water- soluble calcium	24.2	15.1	0.002
3	S-TN	8	3.3	0.082	S-AK	2.9	2	0.148
4	S-AK	4.3	1.9	0.212	S-TP	2.8	1.7	0.2
5	S-TC	3.3	1.3	0.3	S-N:P	2.2	1.6	0.236
6	S-TP	2.9	1.2	0.322	S-TC	1.6	1	0.438
7	S-C:N	0.8	0.3	0.75	S-C:P	0.9	0.6	0.604
8	S-N:P	0.5	0.2	0.838	S-C:N	0.5	0.3	0.734
9	S-C:P	0.1	<0.1	0.962	S-TN	0.4	0.2	0.874

TC, total carbon; TN, total nitrogen; TP, total phosphorus; AK, available potassium.

## 4 Discussion

### 4.1 Effects of exogenous calcium on nutrient uptake and nutrient partitioning in *Pinus tabuliformis* seedlings

Calcium in plants serves not only as a structural component influencing cell wall rigidity, maintaining the stability and permeability of cell membranes, but also as a “second messenger” for intra- and extracellular signaling, responding to various biotic and abiotic stresses, and playing crucial roles in promoting plant growth and nutrient uptake (Liang and Zhang, 2018; Tian et al., 2020; Weng et al., 2022). Previous studies on tall fescue and poplar have shown that the addition of exogenous calcium can promote the absorption and accumulation of C, N, P, K, and Ca by plant organs, and there exists an optimal calcium concentration that maximizes the accumulation of various nutrients (Wang et al., 2017; Weng et al., 2022). Consistent with these findings, the results of this experiment demonstrate that the content of TC, TN, TP, TK, and Ca in different organs of pine seedlings significantly increased after the addition of exogenous calcium, reaching maximum values at calcium concentrations of 50-100 mg·kg^-1^. However, when the calcium concentration exceeds 400 mg·kg^-1^, its nutrient uptake effects are inhibited. This may be attributed to the excessive calcium disrupting normal biochemical and nutritional metabolism in plants, with high concentrations of calcium ions even causing cellular toxicity and abnormal plant development (Li et al., 2023a; Guo et al., 2021).

Calcium utilization efficiency calculated by Chapin’s index showed that the calcium utilization efficiency gradually decreased with the increase of added calcium concentration, which was 85.54% when no calcium was applied, and decreased to 39.78% when the calcium concentration was increased to 400 mg·kg^-1^, which may be due to the abundant supply of calcium nutrient in the soil, and the plant adapted to the environment by reducing the efficiency of the utilization of this nutrient to form the self-regulation mechanism mainly (Chapin, 1980; Liu et al., 2022).The elemental contents of different organs also reflect the physiological activities of plants and their adaptive strategies to the environment. In this study, based on the average proportion of various organs, the following results were obtained: in *Pinus tabuliformis* seedlings, the highest contents of elements C and K were found in the leaves, while elements N, P, and Ca were most abundant in the roots. When calcium was not applied, the total carbon (TC) content in the leaves of *Pinus tabuliformis* seedlings was 524.46 g·kg^-1^, higher than the global average of 464 g·kg^-1^ for plant leaves, with little difference compared to stems (517.37 g·kg^-1^) and higher than roots (317.08 g·kg^-1^) (Elser et al., 2000). This indicates that the ability of carbon fixation in the leaves of *Pinus tabuliformis* seedlings is stronger, resulting in the accumulation of more organic matter, while the storage capacity of roots is weaker. The main reason is that assimilates accumulate more in the leaves and are transported to the stems through conducting tissues, resulting in higher carbon content in stems and leaves, whereas roots mainly exchange water and inorganic salts and cannot photosynthesize (Fan et al., 2019; Huo et al., 2022). Moharam Zadeh et al. (2010) also observed the phenomenon of “most of the plant’s total carbon being stored in the leaves and stems” when evaluating carbon storage in tamarisk. Leaf, stem, and root C contents increased significantly after the application of suitable concentrations of exogenous calcium, which is consistent with the findings of Weng et al. (2022). This may be due to the fact that the applied calcium improved the photosynthetic capacity of the leaves, which in turn increased the accumulation of C in the plant (Song et al., 2020).

### 4.2 Effect of exogenous calcium on ecological stoichiometric ratios of *Pinus tabuliformis* seedlings

The Growth Rate Hypothesis (GRH) suggests that variations in the C:N:P ratio of organisms are mainly determined by changes in the phosphorus content of the biota. Plants with high growth rates require more phosphorus allocated to ribosomes for rapid protein synthesis to support rapid growth, hence they typically have lower C:N and C:P ratios (Reef et al., 2010; Elser et al., 2007). The changing trends of C:N and C:P ratios in *Pinus tabuliformis* seedlings in this study indicate that as calcium concentration increases, the growth rate of *Pinus tabuliformis* seedling roots initially slows down and then accelerates, showing an increasing trend when calcium concentration reaches 400 mg·kg^-1^. The growth rates of leaves, stems, and whole plants also exhibit an accelerating or initially fast and then slow trend. This might be an adaptation to changes in the growth environment. Previous studies by our research group found that when the exogenous calcium concentration exceeds 400 mg·kg^-1^, the *F*_v_/*F*_m_ value of *Pinus tabuliformis* seedlings drops below 0.8, indicating that the plants are under stress and growth and development are severely constrained. Therefore, when calcium concentration is too high, seedlings allocate more nutrients to root growth to avoid stress (Li et al., 2023a). Furthermore, the C:N ratio in *Pinus tabuliformis* seedlings in this study shows a hierarchy of stem (60.86) > leaf (56.65) > whole plant (48.91) > root (35.47), while the C:P ratio shows a hierarchy of leaf (550.38) > whole plant (429.81) > stem (390.05) > root (351.79), all of which are higher than the global average levels of C:N and C:P in plants (22.5 and 232.0) (Elser et al., 2000). This indicates that *Pinus tabuliformis* seedlings have good carbon storage capacity, as well as efficient N and P element utilization, reflecting a relative scarcity of N and P elements required for the growth and development of *Pinus tabuliformis* seedlings in the region (Wei et al., 2021b; Isanta-Navarro et al., 2022). This is consistent with the assertion by Zhang et al. (2018) that the soils in the western Liaoning region are impoverished, with N and P contents below the global average levels. Moreover, the fact that leaf and stem C:N and C:P ratios are higher than those of the roots suggests that the roots have a faster growth rate, which is advantageous for more comprehensive nutrient absorption from deeper soil layers (Ma et al., 2017).

As the exogenous calcium concentration increased, there was no significant difference in the N:P ratio of *Pinus tabuliformis* leaves and stems, but the N:P ratio of roots and the whole plant showed a significant “increase-decrease” trend, which also implies that plants will adjust their stoichiometry to adapt to environmental changes. The N:P ratio within plant tissues reflects environmental factors, especially the nutrient supply from the soil for plant growth, and it can clarify which factors limit plant productivity (Aerts and Chapin, 1999; Güsewell, 2004). Yu et al. (2014) found that when the N:P ratio of needles is< 14, plant growth is limited by N; when N:P = 14-16, plant growth is co-limited by N and P; and when N:P > 16, plant growth is limited by P. In this study, the N:P ratio of *Pinus tabuliformis* seedlings was less than 14, indicating that growth was limited by soil N. Within the calcium concentration range of 50-100 mg·kg^-1^, the N:P ratio was relatively large, indicating that the availability of soil N was higher and the degree of N limitation was relatively light, and as the calcium concentration increased, the limiting effect strengthened, which also indicates that the calcium concentration of 50-100 mg·kg^-1^ was more suitable for the growth of *Pinus tabuliformis*. However, the factors affecting plant N:P values are complex, and it is unreliable to determine the limiting factors in the plant growth process based on a single indicator. Therefore, soil environmental factors should be introduced as auxiliary comprehensive evaluation (Güsewell, 2004). The results of redundancy analysis show that each soil factor has different contribution rates to the various stoichiometric characteristics of *Pinus tabuliformis* seedlings. And previous studies have shown that the absorption of C and other nutrients (P, K, Ca, and Mg) is doubly regulated by stoichiometry and nutrient limitation control, and the nutrient absorption of coniferous trees mainly depends on stoichiometric control rather than nutrient limitation control (Sun et al., 2023).

The stoichiometric steady state in ecology provides a more accurate representation of the physiological and biochemical adaptation of organisms to environmental changes. Research results indicate that there are differences in the elemental dynamic balance indicators and stoichiometric ratios of leaves, stems, roots, and whole plants. This is similar to the findings of Jia et al. (2023), suggesting a trade-off between nutrient uptake and allocation, indicating that the stoichiometric characteristics of plants in soil differ among plant tissues and elemental types. Apart from Ca and some K elements, the internal steady-state model equations did not significantly simulate most indicators (*p* > 0.05) or 1/H< 0, indicating a strong ecological stoichiometric balance with no clear trend in response to changes in soil nutrient environments. This is consistent with the research results of Su et al. (2022); You (2022), and Wang et al. (2022), who observed “strict internal stability” in plants, likely reflecting more on species-specific traits or genetic features (Tao and Zhang, 2015; Erfan et al., 2022). Various factors influence the dynamic balance of ecological stoichiometry, such as soil environmental factors, plant structural characteristics, geography and climate, external disturbances, and human activities (Bai et al., 2019; Zhang et al., 2019). Therefore, continuous monitoring and management of the long-term effects of exogenous calcium on the soil are necessary.

## 5 Conclusion

A well-regulated application of calcium can significantly enhance overall nutrient uptake and accumulation within plants, encompassing their roots, stems, and leaves. The impact of exogenous calcium on plant ecological stoichiometry is greater than that of the soil. This external calcium supplementation facilitates enhanced nutrient absorption and regulates the inter-organ transport of nutrients. Its effectiveness peaks within a specific range, typically between 50-100 mg·kg^-1^. However, excessive exogenous calcium inhibits the absorption of nutrient elements in various plant organs, indicating an adverse calcium stress effect on *Pinus tabuliformis* seedlings. Furthermore, different plant organs exhibit varying stabilities in response to changes in soil conditions, with most indicators demonstrating robust internal stability. Therefore, there exists an optimal calcium concentration for the nutrient uptake and stoichiometric balance of *Pinus tabuliformis* seedlings. The results of this study provide a theoretical basis for future research on *Pinus tabuliformis* plantation forests.

## Data Availability

The raw data supporting the conclusions of this article will be made available by the authors, without undue reservation.
